# Antibodies to Infliximab and Adalimumab in Patients with Rheumatoid Arthritis in Clinical Remission: A Cross-Sectional Study

**DOI:** 10.1155/2015/784825

**Published:** 2015-02-11

**Authors:** Grith P. Eng, Klaus Bendtzen, Henning Bliddal, Michael Stoltenberg, Marcin Szkudlarek, Viktoria Fana, Hanne M. Lindegaard, Emina Omerovic, Pil Højgaard, Elmo K. Jensen, Pierre N. Bouchelouche

**Affiliations:** ^1^Department of Clinical Biochemistry, Copenhagen University Hospital at Køge, 4600 Køge, Denmark; ^2^Department of Rheumatology, Copenhagen University Hospital at Køge, 4600 Køge, Denmark; ^3^The Parker Institute, Department of Rheumatology, Copenhagen University Hospitals at Frederiksberg and Bispebjerg, 2000 Copenhagen F, Denmark; ^4^Institute for Inflammation Research (IIR), Rigshospitalet University Hospital, 2100 Copenhagen, Denmark; ^5^DANBIO registry, Copenhagen Center for Arthritis Research, Center for Rheumatology and Spine Diseases, Copenhagen University Hospital Glostrup, 2600 Glostrup, Denmark; ^6^Department of Rheumatology, Copenhagen University Hospital Glostrup, 2600 Glostrup, Denmark; ^7^Department of Rheumatology, Odense University Hospital, 5000 Odense, Denmark; ^8^Department of Rheumatology, Copenhagen University Hospitals at Frederiksberg and Bispebjerg, 2000 Frederiksberg, Denmark; ^9^Department of Rheumatology, Copenhagen University Hospital at Gentofte, 2900 Gentofte, Denmark; ^10^Department of Rheumatology, Copenhagen University Hospital at Holbæk, 4300 Holbæk, Denmark

## Abstract

*Objective*. To investigate if antibodies towards biological TNF-*α* inhibitors (anti-TNFi Abs) are present in patients with rheumatoid arthritis (RA) in clinical remission and to relate any anti-TNFi Abs to circulating level of TNF-*α* inhibitor (TNFi). *Methods*. Patients with RA, treated with infliximab or adalimumab, and in clinical remission (DAS28(CRP) < 2.6) were included from 6 out-patient clinics. In blood samples, presence of anti-TNFi Abs was determined by radioimmunoassay, and concentration of bioactive TNFi was measured by a cell-based reporter gene assay. *Results*. Anti-TNFi Abs were present in 8/44 patients (18%) treated with infliximab and 1/49 patients (2%) treated with adalimumab (*p* = 0.012). In the former group, anti-TNFi Abs corresponded with low levels of TNFi (*p* = 0.048). Anti-TNFi Ab-positive patients had shorter disease duration at initiation of TNFi therapy (*p* = 0.023) but were similar for the rest of the compared parameters. *Conclusions*. In RA patients in clinical remission, anti-TNFi Abs occur frequently in patients treated with infliximab, while they occur rarely in patients treated with adalimumab. Presence of anti-infliximab Abs is accompanied by low or undetectable levels of infliximab. These data suggest that continued infliximab treatment may be redundant in a proportion of RA patients treated with infliximab and in clinical remission.

## 1. Introduction

Biologic disease-modifying antirheumatic drugs (bio-DMARDs), especially in the form of TNF-*α* inhibitors (TNFi), are established treatment options for patients with a wide variety of immunoinflammatory rheumatic diseases, including rheumatoid arthritis (RA). Since the millennium, knowledge regarding who, when, and how to treat with TNFi has increased greatly, and the aim has moved from suppression of symptoms towards disease control. As biologic medications have potentially life threatening side effects and are expensive, redundant treatment should be avoided.

Some patients develop antibodies against TNFi (anti-TNFi Abs) and most of these antibodies neutralize the effect of the TNFi [1–5]. A relationship has been established between circulating levels of anti-TNFi Abs and low levels of TNFi, as well as impaired clinical response [1–3]. Although the mechanisms underlying this immunological reaction are largely unknown, basic pharmacoimmunological information may be used to improve the use of TNFi therapies. For instance, patients in clinical remission having high levels of anti-TNFi Abs and low levels of TNFi may not benefit from further medication, as maintained remission could be independent of continued TNFi administration [6]. Stopping unnecessary medication would reduce further immunization and the associated safety issues in these individuals and it would greatly reduce the costs of treatment. To estimate the potential of such a strategy, we investigated the presence of antibodies against infliximab and adalimumab, and circulating levels of the corresponding TNFi, in RA patients in clinical remission.

## 2. Patients and Methods

### 2.1. Patients

Through the nationwide DANBIO registry, patients fulfilling the 2010 [7] or 1987 [8] consensus criteria for RA were considered for enrollment in the study. The patients were included from 6 different locations, minimizing geographical selection bias. Inclusion criteria were age of 18 years or older, treatment with infliximab or adalimumab for at least one year, and clinical remission defined as disease activity score in 28 joints (DAS28(CRP)) less than 2.6 [9] at the time of the inclusion. It was not a requirement that patients had to have a shorter or longer history of remission at time of inclusion. Previous therapy with TNFi or other bio-DMARDs did not disqualify inclusion. The 223 patients complying with the inclusion criteria according to DANBIO received a letter with an invitation to study participation. Of these, 111 patients replied positively to the invitation, and a study appointment was made. Eight patients either did not attend this scheduled visit or were excluded at the visit, and following the physical examination and the new DAS28(CRP) based on parameters obtained at the study visit, further 10 patients had to be excluded, leaving 93 patients included.

The study was approved by the local ethics committee of Region Zealand and all patients gave written informed consent prior to inclusion in accordance with the Declaration of Helsinki (http://www.wma.net/en/30publications/10policies/b3/).

### 2.2. Data Collection

The patients were included in the study for one study visit only and were not followed up by the investigators afterwards. The patents continued in their respective out-patient clinic as they had done prior to the study. Disease activity was assessed at inclusion by DAS28(CRP) [10] along with information on concomitant medication. From patient files and from the DANBIO registry, information was obtained from two time points: study inclusion and retrospectively at TNFi initiation. The information included age, gender, duration of disease and clinical remission, TNFi treatment and concomitant medication, and anti-CCP Ab and IgM rheumatoid factor (IgM-RF) status.

Clinical efficacy of TNFi treatment was evaluated retrospectively through DANBIO records by change in DAS28(CRP) from TNFi initiation until registered DAS28(CRP) at the 6-months visit or closest thereafter. EULAR responses were defined as good, moderate or no response (improvement in DAS28(CRP) > 1.2 and DAS28(CRP) ≤ 3.2; improvement in DAS28(CRP) > 1.2 and DAS28(CRP) > 3.2 or improvement in DAS28(CRP) between 0.6 and 1.2 and DAS28(CRP) ≤ 5.1; improvement in DAS28(CRP) < 0.6 or improvement in DAS28(CRP) between 0.6 and 1.2 and DAS28(CRP) > 5.1) [11].

Presence of anti-TNFi Abs and level of TNFi were determined at one time point only, that is, at inclusion in the study and when patients were in remission. Blood samples for the study were drawn as close to the next medication with TNFi as possible in order to assess trough levels of circulating TNFi. In addition to analyses for TNFi and anti-TNFi Abs, sera were analyzed for C-reactive protein (CRP) using a kit for high sensitive CRP (Abbott Laboratories, Copenhagen, Denmark).

### 2.3. Measurements of Serum Levels of TNFi (Infliximab and Adalimumab) and Anti-TNFi Antibodies

Bioactive TNFi levels were measured by reporter gene assays (RGA) (iLite Infliximab Bioassay and iLite Adalimumab Bioassay, resp., Biomonitor, Copenhagen, Denmark) as previously described [12, 13]. The assays for infliximab and adalimumab are based on the human erythroleukemic K562 cell line transfected with an NF*κ*B-regulated firefly luciferase (FL) reporter-gene construct [12]. The cells also contain a renilla luciferase (RL) reporter-gene under the control of a constitutive promoter that allows TNF-*α*-induced FL to be normalized relative to RL expression making results less dependent on cell numbers and differences in cell viability and reducing the influence of serum matrix effects.

TNFi levels were assessed by preparation of increasing concentrations of infliximab or adalimumab in 100 *μ*L of RPMI 1640 in 96-well microtiter plate. These TNFi standards and 100 *μ*L of serially diluted (final 50-fold to 781-fold) serum sample to be tested were preincubated with 2 ng/mL final concentration of human recombinant TNF-*α* (R&D, Minneapolis, MN, USA). After 30 min at 37°C, a human erythroleukemic K562 cell line transfected with an NF*κ*B-regulated firefly luciferase (FL) reporter-gene was added to each well. After 3 hours at 37°C, Dual-Glo Luciferase substrate (Promega, Fitchburg, WI, USA) was added, and the cells were then read for FL activity using a Wallac Victor Light 1420 luminometer (Perkin-Elmer, Waltham, MA, USA). Subsequently, Dual-Glo Stop & Glo reagent (Promega) was added to each well, and the RL activity was read. Normalization was made by dividing the FL with the RL activity in each well. Calculations of the bioactive drug levels in serum were carried out on the basis of the linear part of the calibration curve. Sample concentrations were expressed as drug equivalents in *μ*g/mL.

Anti-infliximab and anti-adalimumab antibodies were measured by fluid-phase radioimmunoassay (RIA) (Biomonitor) as previously described [1, 14]. Anti-TNFi Ab-positivity was defined as detectable or undetectable levels (limit of quantification (LOQ) 10 arbitrary units (U)/mL) of anti-infliximab or anti-adalimumab antibodies.

### 2.4. Statistical Analysis

Differences between treatment groups and anti-TNFi Ab-positive and -negative patients were assessed by the Mann-Whitney test for continuous data or the Fischer exact test for categorical data. Two-sided *p* values less than 0.05 were considered statistically significant. Analyses were performed using the statistical software SAS version 9.2 (SAS Institute Inc., Cary, NC, USA) and GraphPad Prism version 5.04 (GraphPad Software, San Diego, CA, USA). Data are primarily reported as medians (interquartile ranges) or percentages.

## 3. Results

Ninety-three RA patients were included, 44 treated with infliximab and 49 treated with adalimumab. For baseline variables, please see Table 1. The patients had received TNFi for a median of 5 years (IQR 3–7 years) and had been in remission for a median of 17 months (IQR 11–33 months). At TNFi initiation, the median disease duration was 14 years (IQR 8–22 years) and the mean DAS28(CRP) was 4.5 (range 1.4 to 7.5). 25/44 patients on infliximab were treated with standard dose and frequency, 9 with a decrease in dose or frequency, and 10 with an increase in dose or frequency. For adalimumab, 34/49 patients were treated with standard regimen, 13 with reduced frequency, and 2 with increased frequency. The vast majority of patients were also treated with methotrexate from initiation of the biological treatment; 41 of 44 (93%) patients were treated with infliximab and 41 of 49 (84%) were treated with adalimumab. In the infliximab group, one patient started and 3 patients stopped methotrexate between initiation of biologic therapy and sampling for the present study. In the adalimumab group, 5 patients started and 6 patients stopped methotrexate in the same period. All patients developing anti-TNFi Abs had been treated with methotrexate from initiation of TNFi therapy and until sampling for the present study.

For patients treated with adalimumab, a median of 10 days (IQR 6–14 days) had passed from the last administration of adalimumab and until blood samples for the study were drawn. For patients treated with infliximab, a median of 46 days (IQR 34–55 days) had passed.

Anti-TNFi Abs were found in 9 of the included patients (10%): eight of 44 patients (18% (95% CI 7–30%)) treated with infliximab and one of 49 patients (2%) treated with adalimumab (Table 2). The difference in frequency of anti-TNFi Abs between the two drugs was statistically significant.

For the evaluation of TNFi concentration, data from 73 patients were available. The anti-infliximab Ab-positive patients had significantly lower infliximab concentration (median 1.6 *μ*g/mL) compared with the anti-infliximab Ab-negative patients (median 4.7 *μ*g/mL) (Table 3).

Data from 75 patients (39 treated with infliximab and 36 treated with adalimumab) were available for the retrospective evaluation of clinical efficacy of TNFi treatment (Table 4). The change in DAS28(CRP) observed in the first 6–12 months following initiation of TNFi treatment did not differ between the anti-TNFi Ab-positive and -negative patients. Good EULAR response was observed in 58 patients (77%), whereas 4 patients were nonresponders (5%), and the frequency of good EULAR response did not diverge between the anti-TNFi Ab-positive and -negative groups. Patients who later proved anti-TNFi Ab-positive presented with either good or moderate EULAR responses after initiation of TNFi therapy.

At inclusion in the study, the anti-TNFi Ab-positive and -negative patients were similar in regard to age, gender, anti-CCP or IgM rheumatoid factor-positivity, and duration of remission (Table 5).

The anti-TNFi Ab-positive patients had significantly shorter disease duration at TNFi initiation (Table 6). No other parameters investigated differed between the two groups, and none of the anti-TNFi-Ab positive patients had been on a prior bio-DMARD.

## 4. Discussion

In our cohort of RA patients in clinical remission, anti-TNFi Abs were present in a significant percentage of patients receiving infliximab (18%) but only rarely seen in a similar group of adalimumab treated patients (2%). This difference in immunogenicity in the two groups of patients may result from the differences in drug immunogenicity, as infliximab is composed of non-human residues while adalimumab is not. The difference in immunogenicity may also stem from differences in the two patient populations, although we only found a difference in median age at time of inclusion. The observation of patients treated with infliximab being older than patients treated with adalimumab may reflect the practice of prescribing an i.v. administered drug to older patients, and a self-administered s.c. formulation to younger patients. We cannot say if age and age-related factors may influence immunogenicity and development of anti-TNFi Abs.

One would expect that anti-TNFi Ab-positive patients showed less initial improvement, since the presence of anti-TNFi Abs is associated with impaired clinical response [1–3]. This was not the case in our cohort. The frequency of good EULAR response following initiation of therapy did not diverge between the later defined anti-TNFi Ab-positive and -negative groups. Patients who later proved anti-TNFi Ab-positive presented with either good or moderate EULAR responses in the first 6–12 months of treatment. A likely explanation is that anti-TNFi Abs did not form until later in the treatment course, at a time when the patient had already reached TNFi independent remission, and patients with disease relapse would not meet the inclusion criteria of remission for the present study. Of course this is only hypothetical, as we have no information regarding the timing of anti-TNFi Ab development.

The frequency of anti-TNFi Abs in patients with a good clinical response to therapy is in accordance with previous studies. These studies report anti-TNFi Ab development in 4% to 44% of patients in remission as well as with good EULAR response [1–4, 15].

Interestingly, recent evidence suggests that TNF-*α*, as a predominant mediator of chronic inflammatory processes, may fade with time. This could be of potential importance for patients receiving long-term TNFi therapies. For example, some patients with inflammatory bowel disease lose responsiveness to continued infliximab administration in the presence of high levels of TNF-*α* neutralizing activity induced by the previously active drug [14]. If similar changes in pharmacodynamics occur in a subset of patients with RA, that is, that TNF-*α* ceases to contribute to inflammation after prolonged TNFi therapy, these patients might be in remission regardless of receiving sustained TNFi therapy. Indeed, TNF-*α* as a pathogenic factor may have been lost in our anti-infliximab Ab-positive patients since they are in remission despite an assumed Ab-mediated neutralization of the drug during infusions.

Inconsistency in the occurrence of anti-TNFi Abs reported in the literature might be explained by the different techniques used to detect anti-TNFi Abs. Since the discovery of TNFi immunogenicity and anti-TNFi Ab development, reliable detection and quantification of anti-TNFi Abs have become important [16–18]. The most commonly used technique is based on a solid phase, bridging-type enzyme-linked immunosorbent assay (ELISA). Unfortunately, this technique has major limitations for clinical use, as it is unable to detect anti-TNFi Abs in the presence of TNFi. Furthermore, IgG4 anti-TNFi Abs, predominant in long-term immunized patients, also escape detection [16–20]. Solid-phase technologies may also yield false positive and false negative test results due to interference with serum factors and matrix effects. We, therefore, used a more specific and sensitive fluid-phase radioimmunoassay (RIA) for quantification of both anti-infliximab and anti-adalimumab Abs [18]. The RIA for detection of anti-TNFi Abs is the more sensitive method when compared to several other available assays [13]. The impact of residual TNFi on performance of the RIA assay is not known, but it seems that very large concentration of TNFi is needed to completely mask anti-TNFi Abs [1], and it seems that other available assays may be more prone to false negative results on anti-TNFi Ab formation in presence of residual drug [20].

Using a newly developed cell-based assay [12], we found that anti-infliximab Ab-positivity was associated with low serum levels of functionally active drug, suggesting that the antibodies measured by RIA were capable of removing infliximab from the circulation and/or neutralizing drug activity* in vivo*, being of therapeutic significance. It has previously been shown that anti-TNFi Abs bind to the TNF-*α* binding site [5]. The association between anti-infliximab Ab development and low serum levels of infliximab is in accordance with other studies [2, 15, 21]. The single anti-adalimumab Ab-positive patient also exhibited a very low level of adalimumab. Associations between anti-adalimumab Abs, low circulating drug levels, and loss of response have been established in other studies of RA patients [2, 3, 22].

Several previous studies have found an association between the use of concomitant methotrexate and a diminished occurrence of anti-TNFi Abs [3, 23, 24], but other investigators have not found this association [15, 25]. In our study we did not find any association between the use of methotrexate and anti-TNFi Ab development. This could be ascribed to the limited number of anti-TNFi Ab-positive individuals. Alternatively, methotrexate may influence anti-TNFi Ab levels not by its immunosuppressive effect but by its anti-inflammatory effect [17]. This is supported by the fact that all of our patients were in clinical remission. In this case, methotrexate may suppress inflammation (as intended) and thereby aid the TNFi in exerting its effect. Anti-TNFi Abs may be more prone to development if inflammation is insufficiently suppressed. If this is the case, an effect of methotrexate on anti-TNFi Ab levels is only seen in patients with active inflammation, not in patients in long-term remission (the present study). This is supported by the fact that development of anti-TNFi Abs has been associated with lower serum levels of infliximab early in the course of therapy, higher baseline CRP, and higher baseline DAS [1–3, 21].

Contrary to our findings, previous studies have reported longer disease durations in anti-TNFi Ab-positive patients when compared to anti-TNFi Ab-negative patients [3, 26]. The mean disease durations in these studies were approximately eight years, whereas the mean disease duration in our study was 16 years. It must be noted, however, that our cohort selectively consisted of patients in remission, whereas previous studies included patients with ongoing active disease. This difference in patient composition might also explain that the proportion of our patients achieving good EULAR responses is greater than what is generally reported, that is, about one-third [2, 22, 27, 28]. A further explanation for the anti-TNFi Ab-positive patients having shorter disease duration at the initiation of therapy may be that the patients with more active (inflammatory) disease are more likely to be treated with TNFi early in their course of disease. And as previously mentioned anti-TNFi Abs have been associated with higher baseline DAS and CRP.

## 5. Conclusion

To our knowledge, this is the first study to measure anti-TNFi Abs in a cohort of RA patients solely in remission. Despite a median period of remission of 17 months, the patients were still on TNF-*α* inhibitor treatment. We found that 18% (95% CI 7–30%) of infliximab-treated patients in remission had detectable levels of anti-infliximab Abs, and this corresponded to low or absent levels of bioactive infliximab in the blood circulation. We know there is an association between anti-infliximab Abs, low circulating levels of infliximab, and (later) therapeutic failure, and we suspect that TNF-*α*-dependency of chronic inflammation may fade with time in some patients. Hence, we hypothesize that patients achieving remission despite having low drug levels and anti-infliximab Abs may be in remission independent of the use of infliximab. Continued therapy may, therefore, be redundant in these cases. Due to safety issues associated with continuous drug-induced immunization, as well as economic considerations, we suggest further investigations of whether infliximab treatment may be tapered or discontinued in patients with low drug levels and accompanying anti-TNFi Abs. Due to safety issues associated with continuous drug-induced immunization, as well as economic considerations, we suggest further investigations of whether infliximab treatment may be tapered or discontinued in patients with low drug levels and accompanying anti-TNFi Abs, without compromising clinical remission.

According to our data, the issue of antibody formation in RA patients in clinical remission is mainly relevant to infliximab, but it might also be considered in case of long-term remission in patients treated with adalimumab. Future studies may confirm if measurements of anti-TNFi Abs and drug levels will aid in determining when to taper or stop TNFi treatment in RA patients in remission.

## Figures and Tables

**Table 1:** Demographic characteristics of study cohort at time of inclusion and blood sampling.

	Infliximab *n* = 44	Adalimumab *n* = 49	*p*
Age, years	63 (52–71)	54 (46–68)	0.033
Female gender, *n* (%)	27 (61)	28 (57)	0.833
CRP, mg/L	2.3 (1.0–4.2)	1.6 (0.8–3.2)	0.236
DAS28(CRP)	1.6 (1.3–2.0)	1.6 (1.3–2.0)	0.939
Methotrexate use at inclusion, (%)	39 (89)	40 (82)	0.396
Disease duration, years	11 (7–19)	16 (9–23)	0.205
Duration of biologic therapy, months	55 (37–77)	68 (30–93)	0.328
Remission duration, months	17 (11–26)	20 (11–42)	0.609

Continuous variables are expressed as medians (interquartile ranges).

**Table 2:** Anti-TNFi Ab in rheumatoid arthritis patients in remission treated with infliximab or adalimumab.

	Infliximab *n* = 44	Adalimumab *n* = 49	*p*
Anti-TNFi Ab-pos, % (95% CI)	18 (7–30) (*n* = 8)	2 (NA) (*n* = 1)	0.012

**Table 3:** Serum levels of TNF-*α* inhibitor in rheumatoid arthritis patients in remission treated with infliximab or adalimumab.

	Anti-TNFi Ab-negative	Anti-TNFi Ab-positive	*p*
Infliximab, *µ*g/mL	4.7 (2.9–8.0) (*n* = 25)	1.6 (0.7–4.6) (*n* = 6)	0.048
Adalimumab, *µ*g/mL	9.7 (6.5–11.7) (*n* = 41)	0.7 (*n* = 1)	

Continuous variables are expressed as medians (interquartile ranges).

**Table 4:** Initial efficacy (6–12 months) of treatment with infliximab or adalimumab in rheumatoid arthritis patients reaching remission.

	Anti-TNFi Ab-negative *n* = 66	Anti-TNFi Ab-positive *n* = 9	*p*
ΔDAS28(CRP)	2.20 (1.5–3.3)	1.4 (0.9–1.2)	0.212
EULAR good responders, *n* (%)	52 (79)	6 (67)	0.503

Continuous variables are expressed as medians (interquartile ranges); ΔDAS28(CRP): decrease in disease activity score in 28 joints; EULAR: European League Against Rheumatism.

**Table 5:** Characteristics of anti-TNFi Ab-negative and -positive rheumatoid arthritis patients in remission.

	Anti-TNFi Ab-negative *n* = 84 (90%)	Anti-TNFi Ab-positive *n* = 9 (10%)	*p*
Age, years	57 (50–69)	55 (46–66)	0.559
Female gender, *n* (%)	47 (56)	8 (89)	0.077
Seropositivity, *n* (%)	62 (74)	7 (78)	1.000
Duration of biologic therapy, months	60 (35–88)	50 (38–70)	0.413
Remission duration, months	18 (10–33)	17 (16–27)	0.537

Continuous variables are expressed as medians (interquartile ranges).

**Table 6:** Patient characteristics at initiation of TNF-*α* inhibitor therapy and at study inclusion.

	TNFi initiation			Study inclusion		
	Anti-TNFi Ab		*p*	Anti-TNFi Ab		*p*
	Negative	Positive		Negative	Positive	
Disease duration, years	10 (4–18)	2 (2–10)	0.027	15 (8–23)	8 (5–16)	0.045
Methotrexate, *n* (%)	73 (87)	9 (100)	0.250	69 (82)	9 (100)	0.346
Methotrexate, mg/week	15 (7.5–20)	20 (20–22.5)	0.070	10 (7.5–15)	15 (7.5–15)	0.389
Glucocorticoid, *n* (%)	18 (21)	2 (22)	0.979	4 (5)	0	0.506
Glucocorticoid, mg/day	0 (0)	0 (0)	0.822	0 (0)	0	0.993

Continuous variables are expressed as medians (interquartile ranges).
